# α‐Mangostin‐encapsulated PLGA nanoparticles inhibit colorectal cancer growth by inhibiting Notch pathway

**DOI:** 10.1111/jcmm.15731

**Published:** 2020-08-23

**Authors:** Varun Chandra Boinpelly, Raj K. Verma, Sudesh Srivastav, Rakesh K. Srivastava, Sharmila Shankar

**Affiliations:** ^1^ Kansas City VA Medical Center Kansas City MO USA; ^2^ Department of Biostatistics and Data Science School of Public Health and Tropical Medicine Tulane University School of Medicine New Orleans LA USA; ^3^ Stanley S. Scott Cancer Center Louisiana State University Health Sciences Center New Orleans LA USA; ^4^ Department of Genetics Louisiana State University Health Sciences Center New Orleans LA USA; ^5^ John W. Deming Department of Medicine Tulane University School of Medicine New Orleans LA USA

**Keywords:** cancer stem cell, CD133, CD44, colorectal cancer, epithelial‐mesenchymal transition, LGR5, mangostin, musashi, nanoparticles, notch

## Abstract

Colorectal cancer (CRC) is the fourth leading cause of cancer‐related mortality. Recent studies have stated that Notch signalling is highly activated in cancer stem cells (CSCs) and plays an important role in the development and progression of CRC. Like normal colorectal epithelium, CRCs are organized hierarchically and include populations of CSCs. In order to enhance the biological activity of α‐mangostin, we formulated α‐mangostin‐encapsulated PLGA nanoparticles (Mang‐NPs) and examined the molecular mechanisms by which Mang‐NPs inhibit CRC cell viability, colony formation, epithelial‐mesenchymal transition (EMT) and induce apoptosis. Mang‐NPs inhibited cell viability, colony formation and induced apoptosis. Mang‐NPs also inhibited EMT by up‐regulating E‐cadherin and inhibiting N‐cadherin and transcription factors Snail, Slug and Zeb1. As dysregulated signalling through the Notch receptors promotes oncogenesis, we measured the effects of Mang‐NPs on Notch pathway. Mang‐NPs inhibited Notch signalling by suppressing the expression of Notch receptors (Notch1 and Notch2), their ligands (Jagged 1 and DLL4), γ‐secretase complex protein (Nicastrin) and downstream target (Hes‐1). Notch receptor signalling regulates cell fate determination in stem cell population. Finally, Mang‐NPs inhibited the self‐renewal capacity of CSCs, stem cell markers (CD133, CD44, Musashi and LGR5) and pluripotency maintaining factors (Oct4, Sox‐2, KLF‐4, c‐Myc and Nanog). Overall, our data suggest that Mang‐NPs can inhibit CRC growth, EMT and CSCs’ population by suppressing Notch pathway and its target. Therefore, Mang‐NPs can be used for the treatment and prevention of CRC.

## 1 INTRODUCTION

Colorectal cancer (CRC) is the second most common type of malignancy and the fourth leading cause of the cancer‐related death worldwide.^1^ According to American Cancer Society, there will be 104 610 new cases of colon cancer and 43 340 new cases of rectal cancer for 2020 in the United States. The lifetime risk of developing CRC is about 1 in 23 for men and 1 in 25 for women. In United States, it is expected to cause about 53 200 death during 2020. The development of CRC is a complex multistage process which involves sequential mutational events occurring along with progression of the cancer.^2^ Notch and Wnt signalling pathways in CRC development are implicated in the regulation of several biological processes, including cell proliferation, differentiation, angiogenesis, apoptosis and survival.^3^, ^4^ The activation of these pathways is correlated with poor prognosis.^2^, ^5^ Like normal colorectal epithelium, CRCs are organized hierarchically and include populations of CSCs which play significant role in cancer development.^6^, ^7^ Therefore, there is an urgent need to target those pathways which regulate CSCs pool during CRC growth, development and metastasis.

Notch pathway is one of highly conserved cellular pathways responsible for direct cell to cell interaction in multicellular organisms.^8^ Proper function of Notch pathway is essential for normal cell development, differentiation, proliferation and apoptosis.^9^, ^10^, ^11^ Notch signalling pathway consists of five ligands including Jagged‐1, Jagged‐2, Delta‐like‐1 (Dll1), Delta‐like‐3 (Dll‐3) and Delta‐Like‐4 (Dll‐4), and four receptors: Notch1, Notch2, Notch3 and Notch4.^12^ Notch signalling pathway also contains several downstream target genes including Hes‐1, Hey‐1 and p21. While the Notch ligands are the single‐pass transmembrane proteins of DSL family, the Notch receptors are transmembrane proteins containing both types of extracellular and intracellular domains.^12^ Ligand‐binding causes a conformational change leading to ADAM‐mediated ectodomain shedding (S2 cleavage) and subsequent γ‐secretase‐mediated proteolysis within the transmembrane domain (S3/S4 cleavage) resulting in the release Notch intracellular domain (NICD). Subsequently, NICD translocates to the nucleus and associates with the DNA‐binding protein CSL (RBPjκ in mammals) to form a composite interface where the coactivator Mastermind (MAML), p300 and the histone acetyltransferase (HAT) bind, leading to the activation of the transcriptional complex and induction of genes.^13^ Notch signalling mediates the maintenance of intestinal development and homeostasis through the regulation of the differentiation of colonic goblet cells and stem cells/progenitor cells.^14^, ^15^ Notch signalling pathway has a diverse role in tumorigenesis.^16^ Depending on the cellular context, activation of the Notch pathway can exert either an oncogenic or tumour suppressor function.^17^, ^18^, ^19^, ^20^ Moreover, Notch1 plays an oncogenic role in CRC.^4^ The overexpression of Notch1 in CRC cells increased the expression of the downstream targets Hes‐1 and Hey‐1. A recent study has reported that Notch expression in the primary stage of CRC is relatively higher than the later stage. Notch signalling promotes CRC growth through regulation of cell cycle and apoptosis.^21^ Therefore, Notch signalling pathway represents a novel target for cancer therapeutic intervention in CRC.

The development of natural product‐based compounds can be an attractive strategy for the treatment and prevention of CRC because they are non‐toxic, and regulate stem cell population by inhibiting pluripotency and self‐renewal capacity of CSCs via multiple signalling pathways.^22^, ^23^ The α‐mangostin is derived from the plant mangosteen (*Garcinia mangostana*) which was originated in the Sunda Islands and the Moluccas of Indonesia.^24^ Xanthonoid such as α‐mangostin is found in Mangosteen, which grows mainly in Southeast Asia and South America.^25^ Mangostin exerts antioxidant, antimicrobial, anticancer and anti‐inflammatory activities.^26^, ^27^, ^28^, ^29^ Mangostin inhibits mammalian DNA polymerase and topoisomerase activities in cancer cells.^30^ Based on these beneficial effects of mangostin, it can be developed for the treatment and prevention of CRC. Although several studies have demonstrated that α‐Mangostin is safe and well‐tolerated, the in vivo use of α‐mangostin is limited due to its hydrophobic nature, poor aqueous solubility, stability, bioavailability and accumulation in the target organs. To overcome these limitations, we have encapsulated α‐mangostin into the core of poly (D, L‐lactic‐co‐glycolic acid) (PLGA) nanoparticles (Mang‐NPs). Generation of NPs allowed us to increase the efficacy and therapeutic benefits of α‐mangostin for the treatment and prevention of CRC.

The main objective of this paper is to examine the molecular mechanisms by which Mang‐NPs inhibit CRC growth and inhibit the stem cell characteristics. Mang‐NPs inhibited growth of cancer cells and CSCs by suppressing Notch pathway. Mang‐NPs inhibited those genes which play major roles in cell proliferation, self‐renewal, pluripotency, cell cycle, apoptosis and EMT. In conclusion, Mang‐NPs can be used as a potential chemotherapeutic agent for the treatment and prevention of CRC.

## 2 MATERIALS AND METHODS

### 2.1 Reagents

Antibodies against Notch1, Notch2, Jagged1, Hes1, Nicastrin, Dll4, E‐Cadherin, N‐cadherin, Slug, Snail and β‐actin were obtained from Cell Signaling Technology (Danvers, MA). All other chemicals were purchased from Fisher Scientific (Suwanee, GA) and Sigma‐Aldrich (St. Louis, MO).

### 2.2 Production α‐mangostin‐encapsulated PLGA nanoparticles

We have formulated Mang‐NPs as we described earlier.^31^, ^32^ In brief, PLGA (50:50 PLGA, 14 000‐16 000 MW, Sigma‐Aldrich) nanoparticles (NPs) encapsulating α‐mangostin (Mang) were prepared using a double emulsion‐solvent evaporation method. α‐mangostin was purchased from the LKT (St. Paul, MN).

### 2.3 Particle size and zeta potential analysis

Freeze‐dried nanoparticles were suspended in deionized water. The mean particle diameter and width (polydispersity index) were determined by photon correlation spectroscopy using a Zetasizer 3000.^31^, ^32^ The particle charge was quantified as zeta potential by laser Doppler anemometry using the Zetasizer.

### 2.4 Cell culture

Human colorectal cancer cells (HCT116 and HT29) and normal epithelial cells CRL‐1831 were purchased from American Type Culture Collection (ATCC) and maintained in culture conditions as recommended by ATCC (Manassas, VA). We have previously described the isolation and characterization of human CRC CSCs (CD133^+^/CD44^+^/LGR5^+^).^33^ Colorectal CSCs were cultured in stem cell growth medium at 37°C in a humidified atmosphere of 95% air and 5% CO_2_.^34^

### 2.5 Cell viability and apoptosis assays

Cells (1.5 × 10^4^) were grown in cell culture medium and treated with Mang‐NPs (0‐10 µmol/L) for 48 or 72 hours. Viability was measured by CellTiter‐Glo® Luminescent Cell Viability Assay (Promega). The CellTiter‐Glo^®^ Luminescent Cell Viability Assay is a homogeneous method to determine the number of viable cells in culture based on quantitation of the ATP present, which signals the presence of metabolically active cells. The apoptosis was determined by TUNEL assay.^31^, ^35^

### 2.6 Colonosphere (spheroid formation) assay

For colonosphere assay, cells were plated in six‐well ultralow attachment plates (Corning Inc, Corning, NY) at a density of 1000 cells/mL in stem cell growth medium as described.^33^ Human CRC CSCs were treated with Mang‐NPs (0‐10 µmol/L) for 7 days to obtain primary spheroids. Spheroids were collected after 7 days and dissociated with Accutase (Innovative Cell Technologies, Inc). The CSCs obtained from dissociation of spheroids were counted. At the end of incubation period, spheroids were collected, reseeded and treated with Mang‐NPs for another week to obtain secondary spheroids. Secondary spheroids were collected, reseeded and treated with Mang‐NPs for another week to obtain tertiary spheroids. Cell viability in spheroids was measured by trypan blue assay at the end of 7, 14 and 21 days (primary, secondary and tertiary colonosphere, respectively).

### 2.7 Motility assay

Cell motility assay was performed as we described elsewhere.^31^ In brief, cells were grown to a confluent monolayer in a 6‐well plate, scratched with a 200‐μL tip and washed twice with PBS. After incubation with medium containing 1% FBS following treatment with or without Mang‐NPs, the cells were photographed at 0, and 24 hours under an inverted microscope (Olympus, Tokyo, Japan) at 40× magnification. The width of the scratch gap is viewed under the microscope in four separate areas each day until the gap is filled in the untreated control wells.

### 2.8 Transwell migration assay

Transwell migration assays were performed as we described elsewhere.^31^ In brief, 1 × 10^5^ cells in 200 μL of medium with 1% FBS were plated in the top chamber onto the non‐coated membrane (6.5‐mm diameter, 8‐μm pores; Corning Costar, Corning, NY) and allowed to migrate in the lower chamber towards 10% FBS (as chemoattractant)‐containing medium. Cell was treated with Mang‐NPs. After 48 hours of incubation at 37°C in 5% CO_2_, cells were fixed with methanol, stained with crystal violet and counted under an inverted microscope (100 × magnification).

### 2.9 Transwell invasion assay

Transwell invasion assays were performed as we described elsewhere.^31^ In brief, 1 × 10^5^ cells in 200 μL of medium with 1% FBS were plated on top of a layer of Matrigel in transwell chambers (6.5‐mm diameter, 8‐μm pores; Corning Costar, Corning, NY). Cells were treated with Mang‐NPs. Cells were allowed to invade in the lower chamber towards 10% FBS (as chemoattractant)‐containing medium. After 72 hours of incubation at 37°C in 5% CO2, cells that did not migrate were removed from the top of the transwell filters by scraping. The cells that had penetrated the Matrigel were fixed with methanol, stained with crystal violet and counted under an inverted microscope (100 × magnification). The number of penetrated cells represented the invasion activity.

### 2.10 Western blot analysis

Western blot analysis was performed as we described elsewhere.^36^, ^37^ Proteins from the cell lysates were prepared from cells treated with or without Mang‐NPs. Equal amounts of protein were denatured and separated by SDS‐PAGE, transferred onto PVDF membranes and incubated with primary antibody (1:100‐500) followed by secondary antibody (1:5000). The peak intensity of each band was visualized using an Enhanced Chemiluminescence kit (Sigma‐Aldrich).

### 2.11 Quantitative real‐time PCR

Total RNA was extracted from cells using the TRIzol reagent (Invitrogen, Carlsbad, CA, USA) according to the manufacturer's instructions. The concentration, purity and integrity of the RNA were measured using a Nano Drop2000 spectrophotometer (Thermo Scientific). q‐RT‐PCR was performed as described elsewhere.^38^ Briefly, cDNA was synthesized using a high capacity cDNA reverse transcription kit (Applied Biosystems). Primers specific for each of the signalling molecules were designed using NCBI/Primer‐BLAST and used to generate the PCR products. For the quantification of gene amplification, real‐time PCR was performed using an ABI 7300 Sequence Detection System in the presence of SYBR‐Green.

### 2.12 Notch reporter assay

Notch reporter activity was measured as we described elsewhere.^39^, ^40^ In brief, a pGreenFire1‐Notch plasmid that expressed copGFP reporter and firefly luciferase under the control of four Notch response elements and a minimal CMV promoter (pGreen Fire1‐4xNotch‐mCMV‐EF1‐Puro) was purchased from System Biosciences. Lentiviral production and transduction were performed as we described elsewhere.^31^ Infection efficiency of cells with lentivirus was optimized using the positive Lentivirus expressing GFP under the control of CMV promoter, and the MOI was determined to be 10. For transcription assay, cells (10‐40 X 10^3^ cells per well) were seeded in 12‐well plates and treated with Mang‐NPs (0‐10 µmol/L) for up to 48 hours. After incubation, cells were analysed for reporter activity (Promega Corp., Madison, WI).

### 2.13 Immunofluorescence

Colorectal cancer cells were grown in 12‐well plates (Beckton Dickinson, Bedford, MA) and treated with or without a mixture of coumarin‐6 containing Mang‐NPs (5 µmol/L) and DAPI (1 mg/mL) for various time points (0‐24 hours). After washing with PBS, stained cells were visualized under a fluorescent microscope.

### 2.14 Statistical analysis

The mean and SD were calculated for each experimental group. Differences between groups were analysed by ANOVA or t tests using PRISM statistical analysis software (GrafPad Software, Inc, San Diego, CA). Significant differences among groups were calculated at *P* < 0.05.

## 3 RESULTS

### 3.1 Uptake of α‐mangostin‐encapsulated PLGA nanoparticles (Mang‐NPs) by colorectal cancer cells

**Figure 1:**
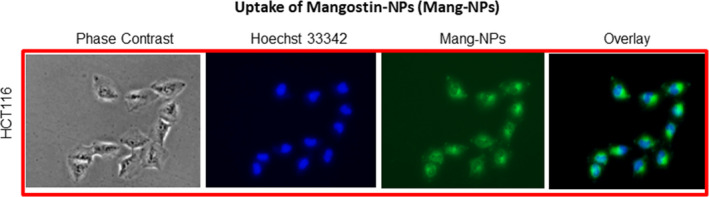
Uptake of α‐Mang‐NPs by colorectal cancer cell line. Colorectal cancer cells (HCT‐116) were treated with coumarin‐6 containing Mang‐NPs for 2 h. Cells were incubated with Hoechst 33 342 for nuclear staining. After washing cells, they were visualized to examine uptake of Mang‐NPs, and photographs were taken by the fluorescence microscope. Green colour = Mang‐NPs. Blue colour = Nucleus

In order to improve the efficacy and improve the half‐life of a drug, nanotechnology has successfully been used.^31^, ^41^ PLGA (50:50 PLGA, 14 000‐16 000 MW, Sigma‐Aldrich) nanoparticles (NPs) encapsulating α‐mangostin and coumarin‐6 (fluorescent dye) were prepared using a double emulsion‐solvent evaporation method as we described elsewhere.^41^ As shown in Figure 1, HCT116 cells were able to absorb Mang‐NPs in 30 minutes as shown by the green fluorescence microscopy. We have also observed the uptake of Mang‐NPs as early as 10 minutes with a maximum fluorescence reaching a maximum at 30 minutes. These data suggest that Mang‐NPs are functional and can enter into the cells.

### 3.2 Mang‐NPs inhibit cell viability and colony formation, and induce apoptosis in colorectal cancer cells, but have no effect on human normal colorectal epithelial cells

**Figure 2:**
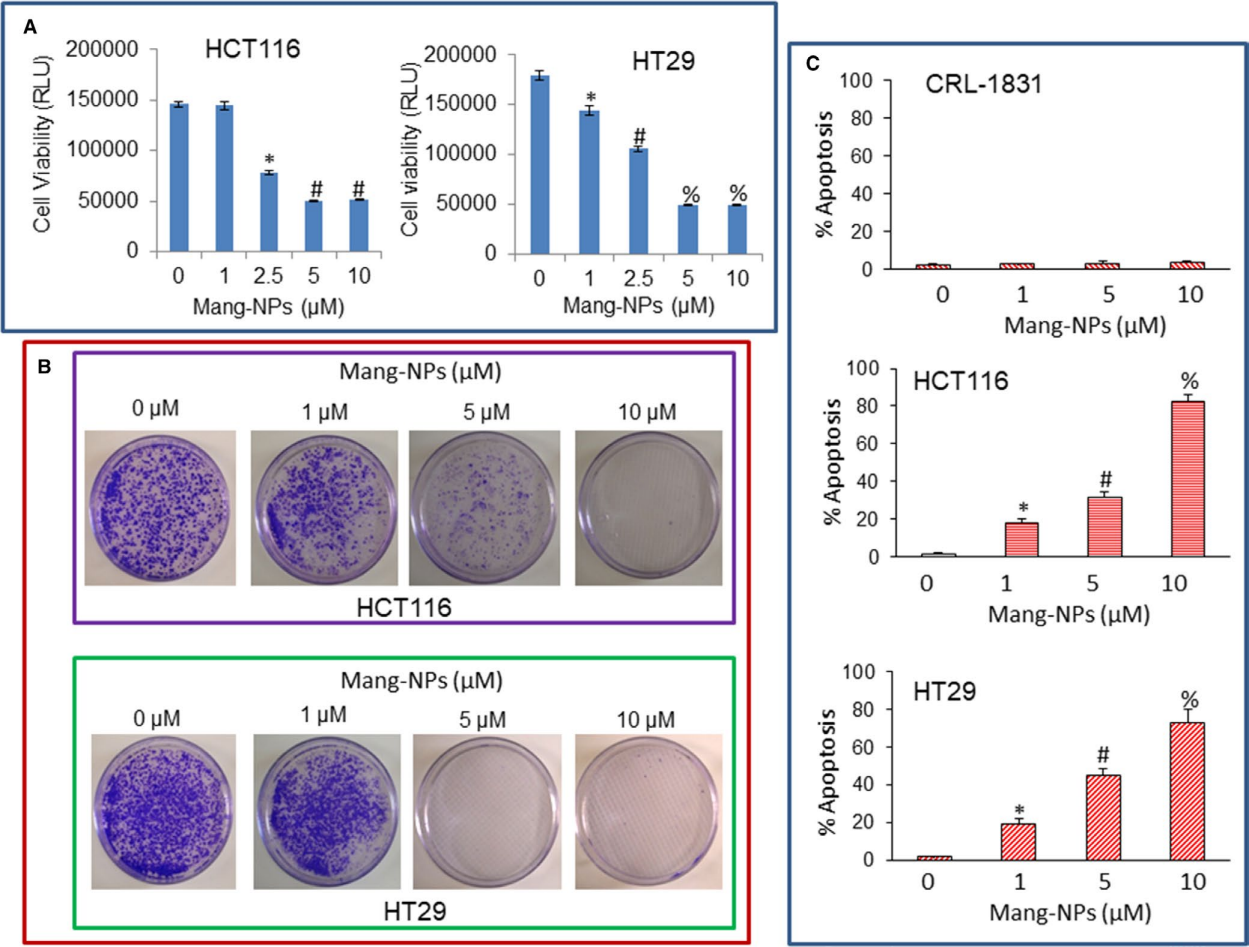
α‐Mang‐NPs inhibit cell proliferation and colony formation, and induce apoptosis in colorectal cancer cells. A, CRC cells were treated with Mang‐NPs (0‐10 µmol/L) for 72 hours, and cell viability was measured as described in Materials and Methods. Data represent mean (n = 4) ± SD. *, #, and % = significantly different from respective control (NPs group), *P* < 0.05. B, HCT‐116 and HT29 cells were treated with NPs or Mang‐NPs (0‐10 µmol/L) for 21 days. Colonies were photographed. C, Colorectal normal CRL‐1831 and cancer cell lines (HT116 and HT29) were treated with Mang‐NPs (0‐10 µmol/L) for 48 hours. Apoptosis was measured by TUNEL assay. Data represent mean ± SD. *, # and % = significantly different from respective control, *P* < 0.05

To assess the efficacy of Mang‐NPs on CRC, we first examined the effects Mang‐NPs on cell viability of CRC HCT116 and HT29 cells using CellTiter‐Glo® Luminescent Cell Viability Assay. Mang‐NPs inhibited cell viability of HCT116 and HT29 cells (Figure 2A). These data suggest that Mang‐NPs can be used as therapeutic agent against CRC.

Colony formation and apoptosis assays are generally used to assess the anticancer activities of antineoplastic drugs. We therefore examined the effects of Mang‐NPs on colony formation and apoptosis. Mang‐NPs inhibited colony formation in CRC HCT116 and HT29 cells in a dose‐dependent manner (Figure 2B). Similarly, Mang‐NPs induced apoptosis in CRC HCT116 and HT29 cells (Figure 2C). Interestingly, Mang‐NPs did not induce apoptosis in human normal colorectal epithelial cells. These data suggest that Mang‐NPs are effective in inhibiting colony formation and inducing apoptosis in CRC cells and can be used as a therapeutic agent.

### 3.3 Mang‐NPs inhibit cell motility, migration and invasion and regulate markers of epithelial‐mesenchymal transition in colorectal cancer cells

**Figure 3:**
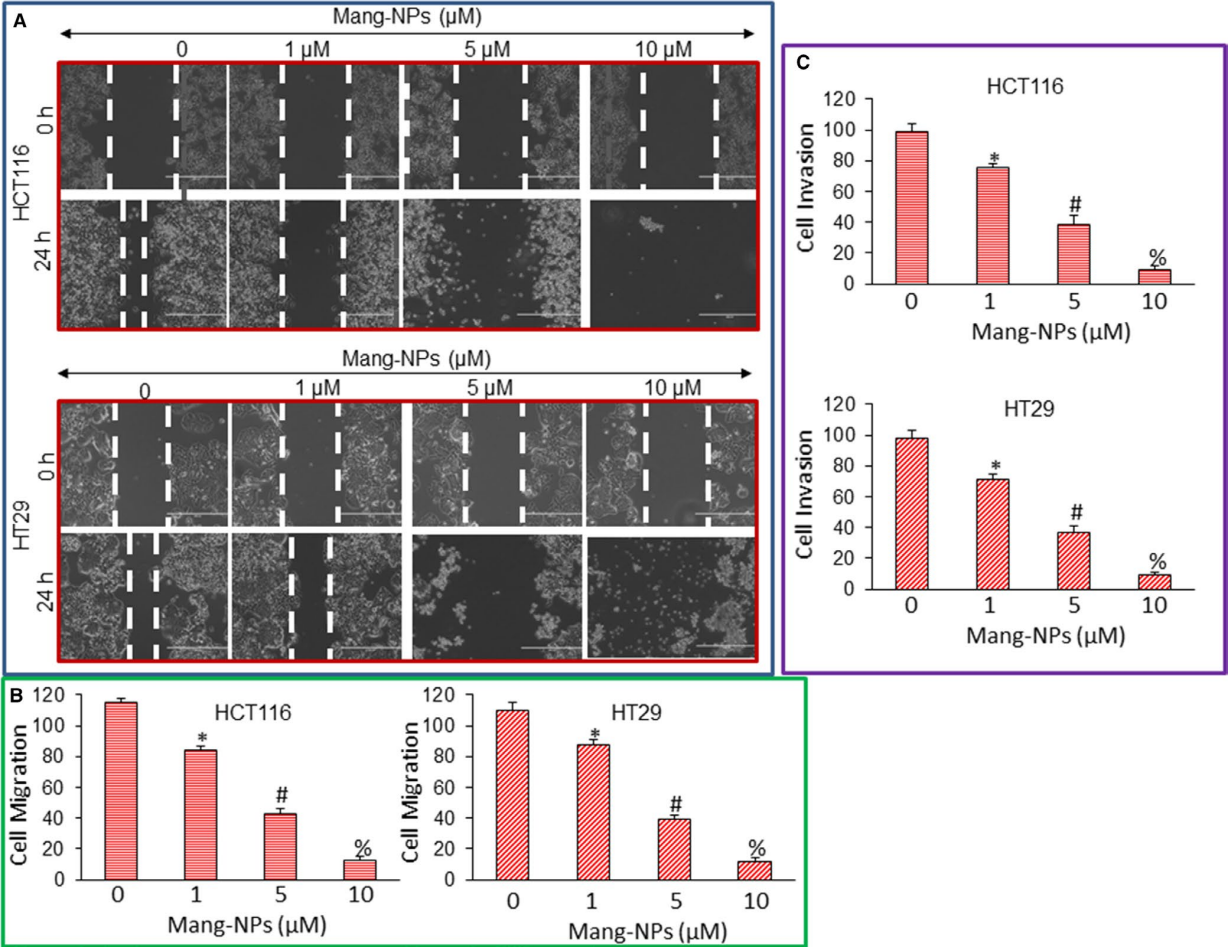
α‐Mangostin inhibits cell motility, migration and invasion and regulates the expression of EMT markers. (A), Cell Motility Assay. Colorectal cancer HCT116 and HT29 cells were grown in monolayer, scratched and treated with or without Mang‐NPs (0‐10 µmol/L) for 24 h. Cells were photographed as we described elsewhere.^49^, ^50^ (B), Cell Migration assay. Colorectal cancer HCT116 and HT29 cells were seeded, treated with Mang‐NPs (0‐10 µmol/L) for 48 h and cell migration assays were performed as described in Materials and Methods. Data represent mean (n = 4) ± SD. *, #, and % = significantly different from control, *P* < 0.05. (C), Cell invasion assay. Colorectal cancer HCT116 and HT29 cells were seeded and treated with Mang‐NPs (0‐10 µmol/L) for 72 h, and cell invasion assays were performed as described in Materials and Methods. Data represent mean (n = 4) ± SD. *, #, and % = significantly different from control, *P* < 0.05

Epithelial‐mesenchymal transition (EMT) is a biological process by which cells undergo through genetic changes that allow them to leave the primary site and migrate to distant location (secondary site) to reestablish, proliferate and survive.^42^ We next measured the effects of Mang‐NPs on cell motility, migration and invasion. Mang‐NPs inhibited cell motility, migration, and invasion of HCT116 and HT29 cells (Figure 3A‐C).

**Figure 4:**
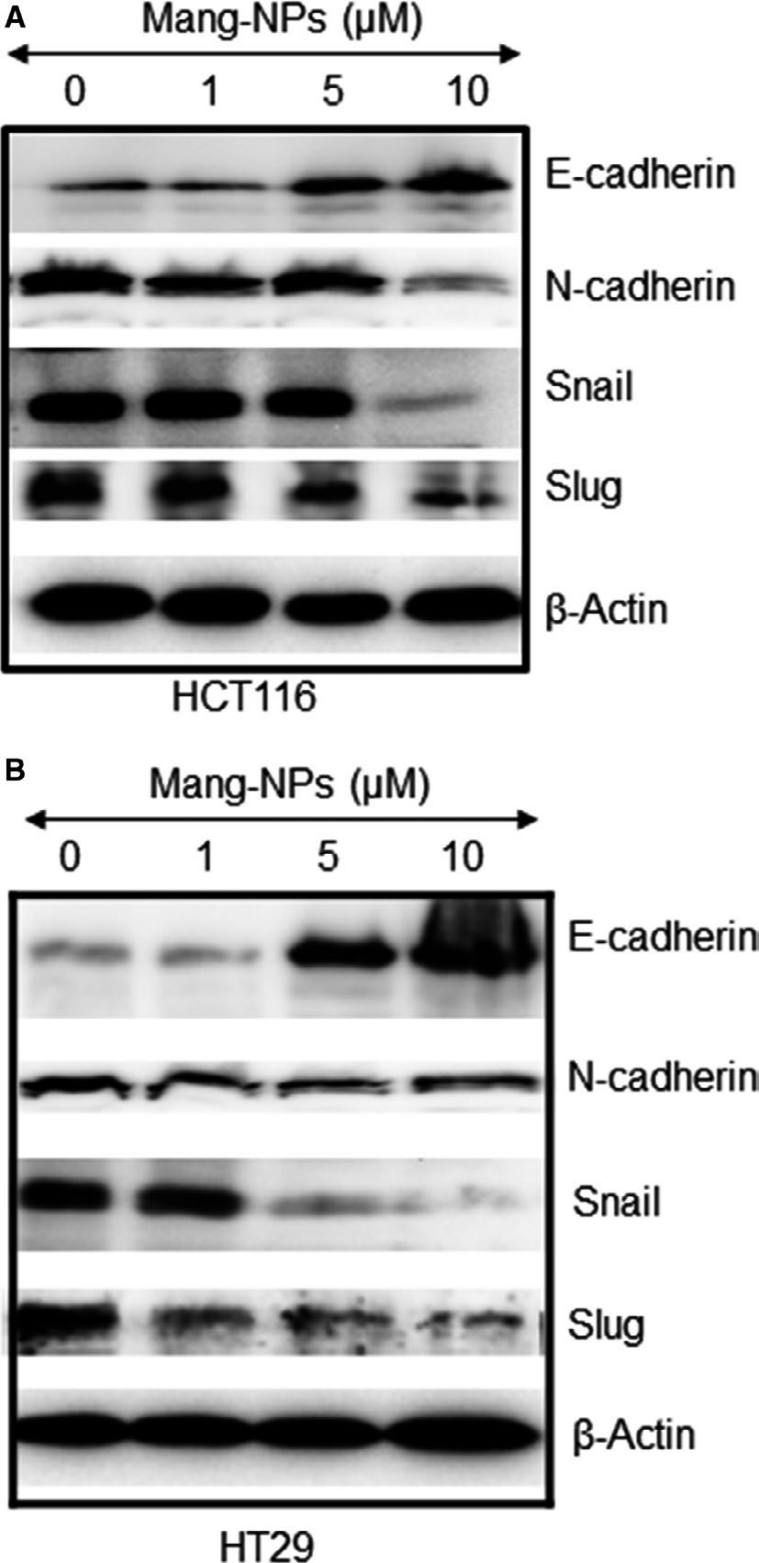
α‐Mangostin regulates the expression of EMT markers. (A and B), Colorectal cancer HCT116 and HT29 cells were treated with or without Mang‐NPs (0‐10 µmol/L) for 48 hours. Western blot analysis was performed to measure the expression of E‐cadherin, N‐cadherin, Snail and Slug. β‐Actin was used as a loading control

As Mang‐NPs inhibited cell motility, migration and invasion, we next sought to examine the molecular mechanisms of EMT regulation by measuring the expression of proteins, that is E‐cadherin, N‐cadherin and transcription factors Snail and Slug. As shown in Figure 4A,B, Mang‐NPs induced the expression of E‐cadherin and inhibited the expression of N‐cadherin, Snail and Slug. These data suggest that Mang‐NPs can inhibit EMT by modulating the expression of cadherins and transcription factors Snail and Slug.

### 3.4 Mang‐NPs inhibit Notch signalling pathway and target proteins in colorectal cancer cells

**Figure 5:**
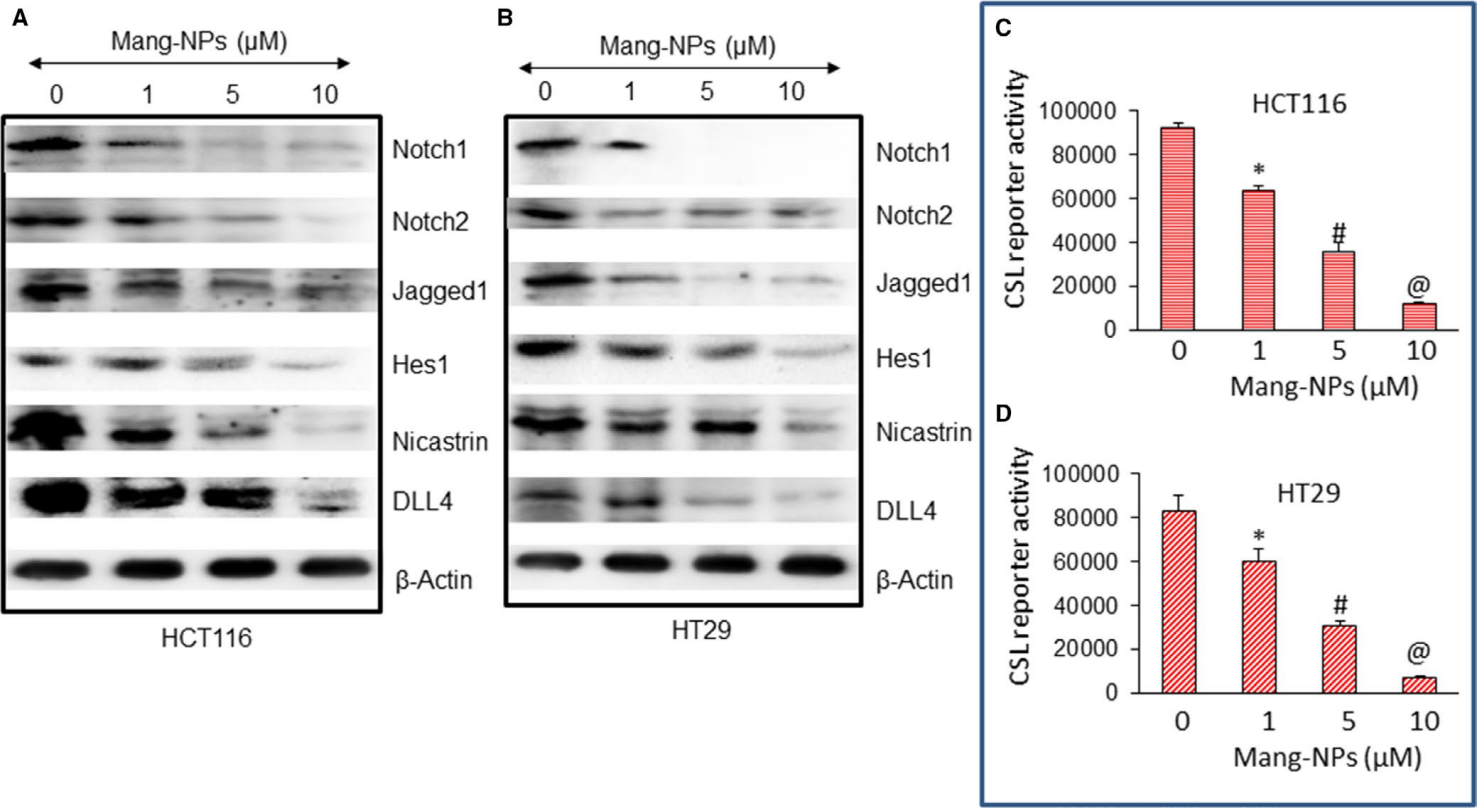
Regulation of Notch pathway and CSL transcription by α‐Mang‐NPs. (A), HCT116 cells were treated with Mang‐NPs (0‐10 µmol/L) for 48 h. The expression of Notch1, Notch2, Jagged1, Hes1, Nicastrin and DLL4 was measured by the Western blot analysis. β‐actin was used as a loading control. (B), HT29 cells were treated with Mang‐NPs (0‐10 µmol/L) for 48 h. The expression of Notch1, Notch2, Jagged1, Hes1, Nicastrin and DLL4 was measured by the Western blot analysis. β‐actin was used as a loading control. (C and D), Regulation of CSL transcription by Mang‐NPs. HCT116 and HT29 cells were transduced with Notch‐responsive GFP/firefly luciferase viral particles (pGreen Fire1‐Notch with EF1, System Biosciences). Transduced cells were treated with Mang‐NPs (0‐10 µmol/L) for 24 h. CSL reporter activity was measured as we described.^40^ Data represent mean ± SD. *, # and @ = significantly different from control, *P* < 0.05

As Notch signalling pathway plays an oncogenic role in CRC, we sought to measure the effects of Mang‐NPs on the components of Notch pathway and downstream targets. Mang‐NPs inhibit the expression of Notch1, Notch2, Jagged1, Hes1, Nicastrin and Dll4 in CRC HCT116 and HT29 cells (Figure 5A‐B). These data suggest that Mang‐NPs can inhibit CRC growth by targeting Notch pathway.

Activation of Notch pathway finally results in CSL transcription and target gene induction; therefore, we next measured the effects of Mang‐NPs on Notch transcriptional activity in HCT116 and HT29 cells by reporter assay. Mang‐NPs inhibited Notch reporter activity in CRC cell lines in a dose‐dependent manner (Figure 5C‐D). These data suggest that Mang‐NPs can inhibit cell proliferation of CRC cells by inhibiting Notch pathway and its target.

### 3.5 Mang‐NPs inhibit cell viability in spheroids, stem cell markers and pluripotency maintaining factors in colorectal CSCs

**Figure 6:**
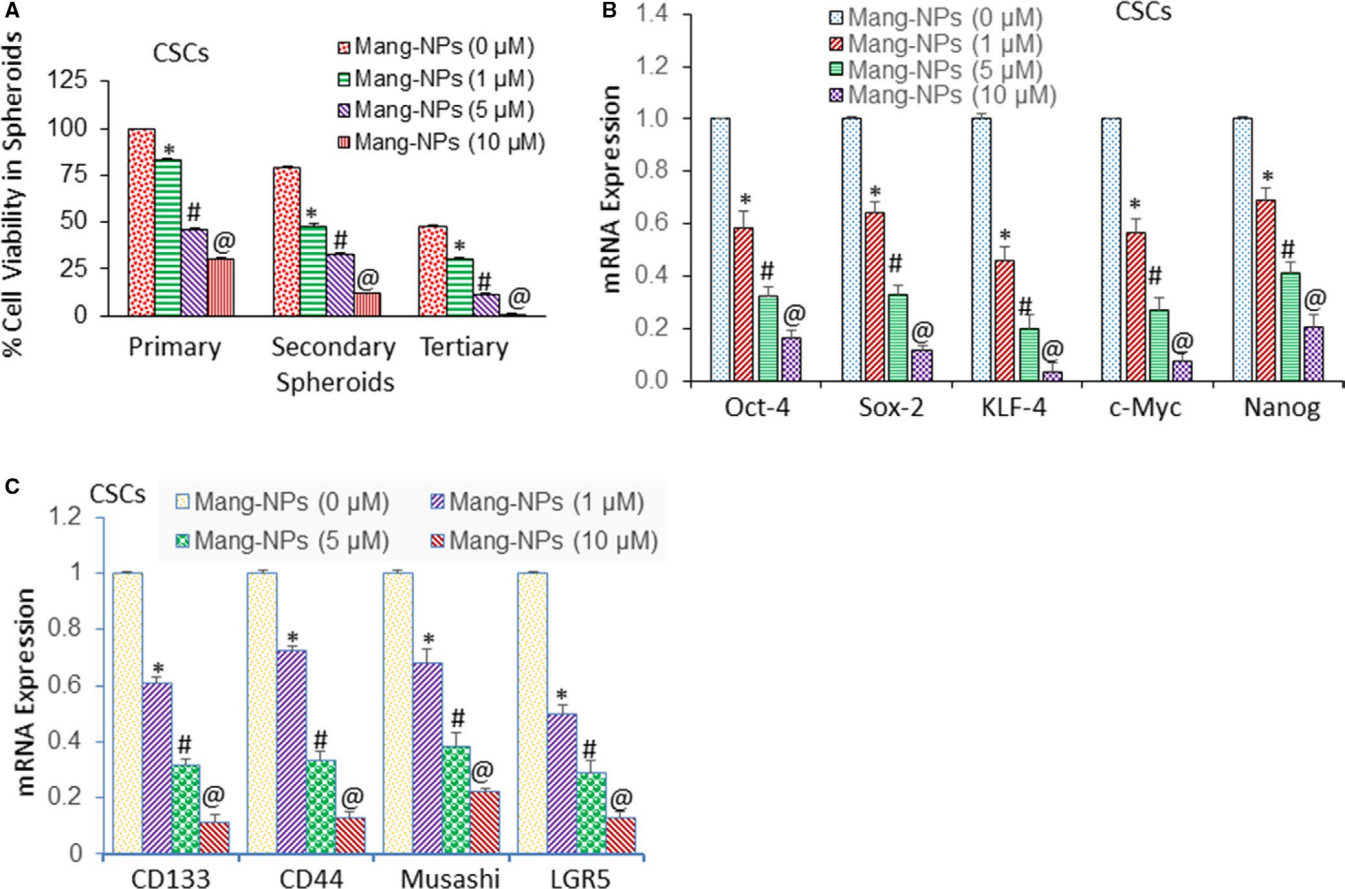
α‐Mang‐NPs inhibit cell viability in spheroids formed by CSCs isolated from human CRC tissues, and expression of stem cell markers and pluripotency maintaining factors. (A), Human colorectal CSCs were treated with Mang‐NPs (0‐10 µmol/L) for 7 days to obtain primary spheroids. At the end of incubation period, spheroids were collected, reseeded and treated with Mang‐NPs for another week to obtain secondary spheroids. Secondary spheroids were collected, reseeded and treated with Mang‐NPs for another week to obtain tertiary spheroids. Cell viability in spheroids was measured by trypan blue assay at the end of 7, 14 and 21 days. Data represent mean ± SD. *, # and @ = significantly different from control, *P* < 0.05. (B), Expression of stem cell markers. Colorectal CSCs were treated with Mang‐NPs (0‐10 µmol/L) for 36 h. The expression of CD133, CD44, Musashi and LGR5 was measured by q‐RT‐PCR. Data represent mean ± SD. *, # and @ = significantly different from control, *P* < 0.05. (C), Expression of pluripotency maintaining factors. Colorectal CSCs were treated with Mang‐NPs (0‐10 µmol/L) for 36 h. The expression of Oct‐4, Sox‐2, KLF‐4, c‐Myc and Nanog was measured by q‐RT‐PCR. Data represent mean ± SD. *, # and @ = significantly different from control, *P* < 0.05

As CSCs play a major role in colorectal carcinogenesis and are responsible for cancer initiation, progression, metastasis and drug resistance, they can be used to evaluate the efficacy of anticancer drugs. Spheroid formation in suspension has been used to measure the stem cell characteristics in vitro. Mang‐NPs inhibited cell viability of primary, secondary and tertiary spheroids formed by colorectal CSCs isolated from primary tumours (Figure 6A). These data suggest that Mang‐NPs can be used for the treatment of CRC because they target self‐renewal capacity of CSCs.

As Mang‐NPs inhibited spheroid formation, we next sought to measure its effects on the expression of commonly used CRC stem cell markers CD133, CD44, Musashi and LGR5 by q‐RT‐PCR. As shown in Figure 6B, Mang‐NPs inhibited the expression of CD133, CD44, Musashi and LGR5. We next measured the expression of pluripotency maintaining factors Oct‐4, Sox‐2, KLF‐4, c‐Myc and Nanog by q‐RT‐PCR. These factors are required for maintaining pluripotency and self‐renewal of CSCs. Mang‐NPs inhibited the expression of Oct‐4, Sox‐2, KLF‐4, c‐Myc and Nanog in a dose‐dependent manner (Figure 6C). These data suggest that Mang‐NPs can inhibit CSC characteristics and stem cell population in CRC.

### 3.6 Mang‐NPs inhibit cell motility, migration and invasion and markers of epithelial‐mesenchymal transition in colorectal CSCs

**Figure 7:**
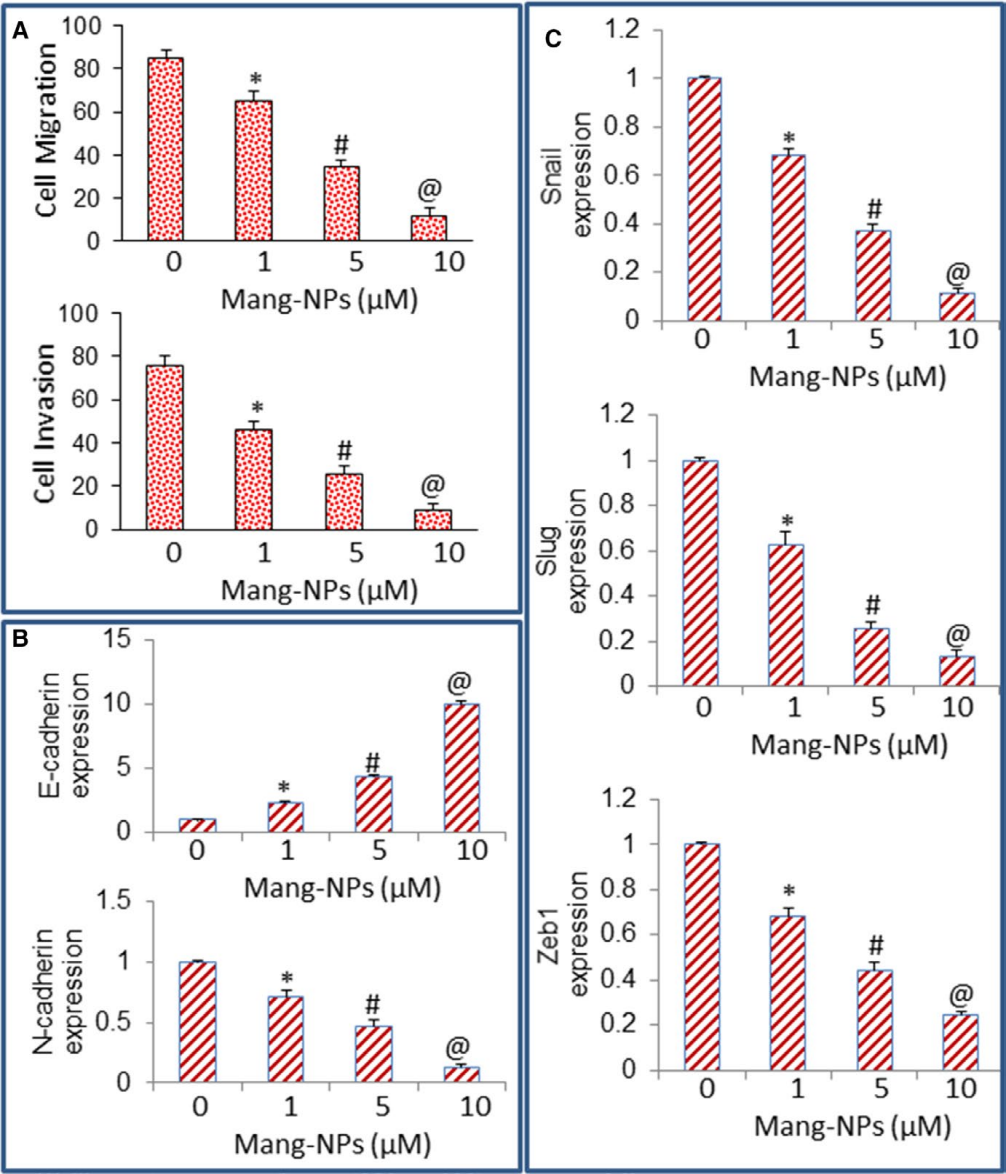
Regulation of cell migration, invasion and expression of EMT‐related transcription factors. (A), Cell Migration and Invasion assays. Colorectal CSCs were seeded and treated with Mang‐NPs (0‐10 µmol/L) for 48 h (migration) or 72 h (invasion), and cell migration and invasion assays were performed as described in Materials and Methods. Data represent mean (n = 4) ± SD. *, #, and @ = significantly different from control, *P* < 0.05. (B), Expression of E‐cadherin and N‐cadherin. CSCs were treated with Mang‐NPs (0‐10 µmol/L) for 36 h. The expression of E‐cadherin and N‐cadherin was measured by q‐RT‐PCR. Data represent mean ± SD. *, # and @ = significantly different from control, *P* < 0.05. (C), Expression of Snail, Slug and Zeb1. CSCs were treated with Mang‐NPs (0‐10 µmol/L) for 36 h. The expression of Snail, Slug and Zeb1 was measured by q‐RT‐PCR. Data represent mean ± SD. *, # and @ = significantly different from control, *P* < 0.05

During epithelial‐mesenchymal transition (EMT), cells undergo through genetic changes that allow them to leave the primary site and migrate to distant location where they reestablish, create a suitable environment and proliferate.^42^ Therefore, we next measured the effects of Mang‐NPs on migration and invasion of CSCs, and measured its effects on the expression of EMT markers and transcription factors. Mang‐NPs inhibited migration and invasion of colorectal CSCs (Figure 7A).

As Mang‐NPs inhibited migration and invasion of CSCs, we next examined the molecular mechanisms of EMT regulation by measuring the expression of genes, E‐cadherin, N‐cadherin and transcription factors Snail, Slug and Zeb1. Mang‐NPs induced the expression of E‐cadherin and inhibited the expression of N‐cadherin (Figure 7B). We next measured the EMT‐related transcription factors such as Snail, Slug and Zeb1 by reporter assay. Mang‐NPs inhibited the expression of Snail, Slug and Zeb1 in colorectal CSCs (Figure 7C). These data suggest that Mang‐NPs are capable of inhibiting EMT and thus may be useful in suppressing metastasis which is predominant in CRC.

### 3.7 Mang‐NPs inhibit Notch signalling pathway and target genes in colorectal CSCs

**Figure 8:**
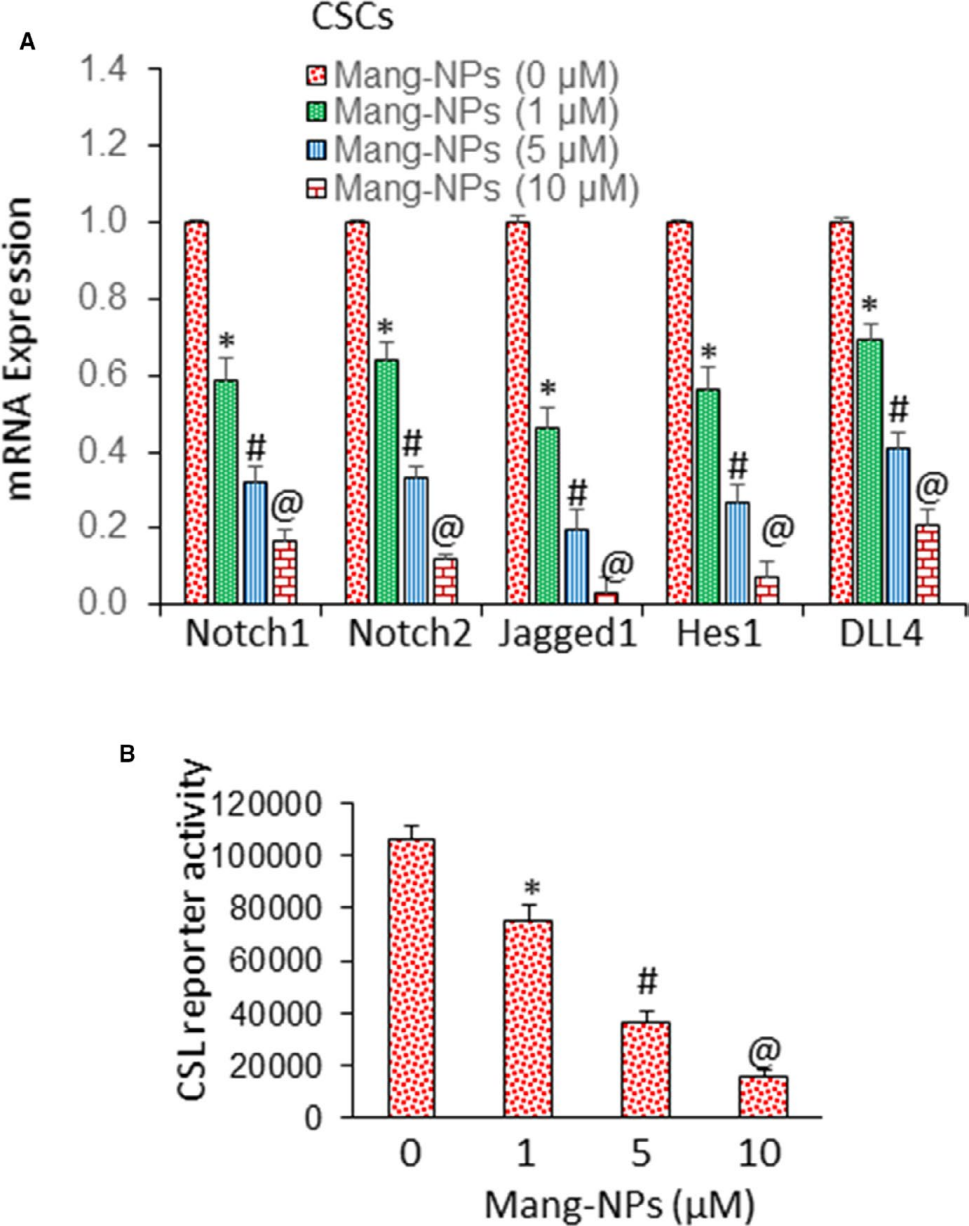
Regulation of Notch pathway and CSL transcription by α‐Mang‐NPs. (A), Colorectal CSCs were treated with Mang‐NPs (0‐10 µmol/L) for 36 h. The expression of Notch1, Notch2, Jagged1, Hes1 and DLL4 was measured by the q‐RT‐PCR. Data represent mean ± SD. *, # and @ = significantly different from control, *P* < 0.05. (B), Regulation of CSL transcription by Mang‐NPs. CSCs cells were transduced with Notch‐responsive GFP/firefly luciferase viral particles (pGreen Fire1‐Notch with EF1, System Biosciences). Transduced cells were treated with Mang‐NPs (0‐10 µmol/L) for 24 h. CSL reporter activity was measured as we described elsewhere.^40^ Data represent mean ± SD. *, # and @ = significantly different from control, *P* < 0.05

Notch pathway regulates stem cell characteristics and plays significant role in CRC carcinogenesis.^43^ We therefore sought to examine the effects of Mang‐NPs on the components of Notch pathway and its downstream targets. Mang‐NPs inhibited the expression of Notch1, Notch2, Jagged1, Hes1 and Dll4 in CSCs isolated from primary CRC (Figure 8A). As activation of Notch pathway regulates CSL transcription, we next measured the effects of Mang‐NPs on CSL transcriptional activity (Figure 8B). These data suggest that Mang‐NPs can inhibit self‐renewal capacity of colorectal CSCs by targeting Notch pathway and its target.

## 4 DISCUSSION

Our data demonstrate that Mang‐NPs can inhibit growth of colorectal cancer cells by suppressing Notch signalling pathway. Mang‐NPs can be easily taken up by cancer cells. Mang‐NPs inhibit cell viability, colony formation and EMT, and induce apoptosis in cancer cells without affecting colorectal normal epithelial cells. In addition to inhibiting growth of CRC cells, Mang‐NPs also inhibited self‐renewal capacity of CSCs by suppressing Notch signalling pathway, stem cells markers and pluripotency maintaining factors. Mang‐NPs inhibit EMT by inducing cadherin switch and suppressing the expression of EMT transcription factors in CSCs. Our findings suggest that Mang‐NPs can be a useful therapeutic agent because it inhibits the growth of not only CRC cells but also CSCs which play major roles in cancer progression, metastasis and drug resistance. Furthermore, our novel findings provide a new avenue to target a stemness‐associated signalling axis as a therapeutic strategy to reduce metastatic spread and cancer recurrence.

Aberrant activation of Notch signalling pathway has been implicated in CRC. The activation of Notch pathway plays a vital role in the progression of CRC. Notch promotes stemness and EMT, and is required for repression of secretory cell differentiation in CRC.^44^, ^45^ In addition, Notch pathway activation was also shown to drive chemoresistance in cancer. The combination of chemotherapy with NOTCH1 inhibitor synergistically attenuated chemotherapy‐enriched CSC population.^46^, ^47^ In the present study, we have demonstrated that Mang‐NPs inhibited Notch signalling by reducing the expression of Notch receptors (Notch1 and Notch2), their ligands (Jagged 1 and DLL4), γ‐secretase complex protein (Nicastrin) and downstream target Hes‐1. These data suggest that Mang‐NPs can inhibit CRC carcinogenesis by inhibiting cell survival, proliferation and cell cycle through Notch pathway.

Cancer stem cells have been implicated in cancer progression, metastasis, drug resistance and cancer relapse. They reside within tumour mass and possess the capacity to self‐renew and to cause the heterogeneous lineages of cancer cells that comprise the tumour. Therefore, eradicating the CSCs and recurrence is considered as a promising strategy to cure the cancer. In the present study, Mang‐NPs inhibited the expression of stem cells markers (CD133, CD44, Musashi and LGR5) and pluripotency maintaining factors (Oct‐4, Sox‐2, KLF‐4, c‐Myc and Nanog) in CSCs, suggesting that Mang‐NPs can inhibit CSC characteristics and stem cell population in CRC. The CSCs like any other stem cells are activated by the signal transduction pathways that are involved in the development and tissue homeostasis. The new treatments targeting signalling pathways, control stem cell replication, survival and differentiation, are under intense investigation. Notch inhibitors either single or in combination with chemotherapy drugs are being developed to treat cancer and its recurrence. It is also important to consider that CSCs are regulated by several additional factors, including genetic alterations, the ECM niche microenvironment, micro RNA's, hedgehog and Wnt pathways, and cycling state (quiescent vs. active state). Altogether the nature of drug resistance of CSCs is multifactorial, various signalling pathways with complex mechanisms could fine‐tune chemosensitivity. The approach of targeting signalling pathway of CSCs by nanotechnology represents a promising future direction for the therapeutic strategy to cure cancer.

Nanotechnology has been successfully used to deliver anticancer drugs because nanoparticles enhance the bioavailability and distribution, preserve the integrity of compounds and improve the biological activity of drugs. In the present study, Mang‐NPs inhibited CRC growth and CSC characteristics by suppressing Notch signalling pathway. In another studies, we have demonstrated that Mang‐NPs and Antho‐NPs (anthothecol‐encapsulated NPs) suppressed proliferation of pancreatic CSCs and cancer cells, and inhibited the self‐renewal capacity of CSCs isolated from pancreatic cancer tissues from human and Kras^G12D^ mice.^31^, ^32^ Similarly, α‐Mangostin‐encapsulated Gold/polyethyleneimine/cyclodextrin (AuNPs/PEI/CD) nanoparticles inhibited prostate cancer growth.^48^ Clinical trials are needed to demonstrate the safety and efficacy of Mang‐NPs for the treatment and prevention of solid tumours.

In conclusion, our study has demonstrated that α‐Mang‐NPs can inhibit the growth of CRC and self‐renewal capacity of CSCs. As α‐Mang‐NPs inhibited stem cell markers and pluripotency maintaining factors, it offers a great potential to inhibit CRC growth and metastasis by targeting CSC population. Therefore, α‐Mang‐NPs offer new hope for the treatment and/or prevention of CRC.

## CONFLICT OF INTEREST

VCB, RKV and SS have declared that no competing interests exist. RKS and SS have declared intellectual property interest.

## AUTHOR CONTRIBUTION

**Varun Chandra Boinpelly:** Conceptualization (equal); Data curation (equal); Formal analysis (equal); Investigation (equal); Methodology (equal); Project administration (equal); Resources (equal); Validation (equal); Visualization (equal); Writing‐original draft (equal). **Raj J Verma:** Conceptualization (equal); Data curation (equal); Formal analysis (equal); Investigation (equal); Methodology (equal); Project administration (equal); Validation (equal); Writing‐original draft (equal). **Sudesh Srivastav:** Methodology (equal); Visualization (equal). **Rakesh K. Srivastava:** Supervision (equal). **Sharmila Shankar:** Supervision (equal).

## Data Availability

The data that support the findings of this study are available from the corresponding author upon reasonable request.
